# PET Imaging of Soluble Yttrium-86-Labeled Carbon Nanotubes in Mice

**DOI:** 10.1371/journal.pone.0000907

**Published:** 2007-09-19

**Authors:** Michael R. McDevitt, Debjit Chattopadhyay, Jaspreet S. Jaggi, Ronald D. Finn, Pat B. Zanzonico, Carlos Villa, Diego Rey, Juana Mendenhall, Carl A. Batt, Jon T. Njardarson, David A. Scheinberg

**Affiliations:** 1 Molecular Pharmacology and Chemistry Department, Departments of Medicine, Radiology, and Medical Physics, Memorial Sloan-Kettering Cancer Center, New York, New York, United States of America; 2 Department of Biomedical Engineering, Cornell University, Ithaca, New York, United States of America; 3 Department of Food Science, Cornell University, Ithaca, New York, United States of America; 4 Department of Chemistry and Chemical Biology, Cornell University, Ithaca, New York, United States of America; National Institutes of Health, United States of America

## Abstract

**Background:**

The potential medical applications of nanomaterials are shaping the landscape of the nanobiotechnology field and driving it forward. A key factor in determining the suitability of these nanomaterials must be how they interface with biological systems. Single walled carbon nanotubes (CNT) are being investigated as platforms for the delivery of biological, radiological, and chemical payloads to target tissues. CNT are mechanically robust graphene cylinders comprised of sp^2^-bonded carbon atoms and possessing highly regular structures with defined periodicity. CNT exhibit unique mechanochemical properties that can be exploited for the development of novel drug delivery platforms. In order to evaluate the potential usefulness of this CNT scaffold, we undertook an imaging study to determine the tissue biodistribution and pharmacokinetics of prototypical DOTA-functionalized CNT labeled with yttrium-86 and indium-111 (^86^Y-CNT and ^111^In-CNT, respectively) in a mouse model.

**Methodology and Principal Findings:**

The ^86^Y-CNT construct was synthesized from amine-functionalized, water-soluble CNT by covalently attaching multiple copies of DOTA chelates and then radiolabeling with the positron-emitting metal-ion, yttrium-86. A gamma-emitting ^111^In-CNT construct was similarly prepared and purified. The constructs were characterized spectroscopically, microscopically, and chromatographically. The whole-body distribution and clearance of yttrium-86 was characterized at 3 and 24 hours post-injection using positron emission tomography (PET). The yttrium-86 cleared the blood within 3 hours and distributed predominantly to the kidneys, liver, spleen and bone. Although the activity that accumulated in the kidney cleared with time, the whole-body clearance was slow. Differential uptake in these target tissues was observed following intraveneous or intraperitoneal injection.

**Conclusions:**

The whole-body PET images indicated that the major sites of accumulation of activity resulting from the administration of ^86^Y-CNT were the kidney, liver, spleen, and to a much less extent the bone. Blood clearance was rapid and could be beneficial in the use of short-lived radionuclides in diagnostic applications.

## Introduction

How nanomaterials interface with biological systems is a key question that may impact the emerging field of nanomedicine. The pharmacokinetic profile of these unique, new materials is a prominent factor in determining their suitability for *in vivo* applications. The important issues of where they distribute *in vivo* and how they clear from a living system must be addressed. Single walled carbon nanotubes [1] (CNT) are promising scaffolds for transporting biological cargo across cellular membranes [2]–[5]. A report [6] of the murine biodistribution of an ^125^I-labeled hydroxylated-CNT into the stomach, kidney, and bone was contrasted by another report [7] describing the rapid blood and whole body clearance of an ^111^In-labeled diethylenetriaminepentaacetic acid (DTPA) derivatized-CNT from mice. The pharmacokinetics of an unmodified-CNT suspended in surfactant was also reported [8] in rabbits utilizing the inherent CNT near-infrared fluorescence for detection and demonstrated rapid blood clearance and only liver accumulation. We attached 1,4,7,10-tetraazacyclododecane-1,4,7,10-tetraacetic acid (DOTA), a radiometal chelate moiety, to soluble, amino-functionalized-CNT [9], [10] and radiolabeled with the short half-lived, positron-emitting ^86^Y (t_1/2_ = 14.7 h) or the long half-lived, gamma-emitting ^111^In (t_1/2_ = 2.81 d). The goal of this study was to map the *in vivo* distribution of our soluble prototype CNT scaffold in real time and evaluate the pharmacokinetic profile using PET and conventional tissue harvest techniques. Herein, we report the PET imaging and biodistribution of a positron-emitting CNT construct in mice. PET was chosen because it provides extremely sensitive, quantitative, and functional information that is different from that obtainable with other largely anatomical imaging modalities and CT was performed to confirm anatomical assignments [11].

## Methods

### Synthesis and Characterization of ^86^Y-CNT

**Figure 1:**
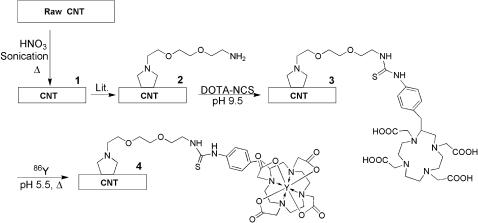
Synthetic scheme to prepare ^86^Y-CNT.

Pristine single walled carbon nanotubes (CNT) were shortened and purified by oxidative acid digestion. Briefly, 60 mg of CNT (Nanostructured & Amorphous Materials, Los Alamos, NM, Lot # 1280YJ) were refluxed in 2M HNO_3_ (60 mL) for 48 hours. In addition, sonication was performed at 70°C with 100 watts for 0.5 h with a Biologics Model 300 V/T Ultrasonic Homogenizer (Manassas, VA) using the 3/8” diameter stepped titanium tip. The CNT were again refluxed for an additional 10 h in the 2M HNO_3_. This digestion and sonication yields purified and shortened carboxy-functionalized CNT molecules (Figure 1, Compound **1**). Raman spectroscopy was employed to characterize **1** using a Renishaw InVia microRaman system (Gloucestershire, United Kingdom) equipped with a 488 nm laser. Samples were evaporated on silicon substrates.

The carboxy-functionalized CNT molecules were functionalized and subsequently solublized by covalent attachment of reactive sidewall amino groups [9], [10] yielding CNT-(NH_2_) (Figure 1, compound **2**). Raman spectroscopy was employed to characterize **2** (as described above) and the number of primary amines that were appended per mass of CNT was measured using the Sarin assay [12].

The water soluble CNT-amine construct, **2**, was reacted with 2-(p-isothiocyanatobenzyl)-1,4,7,10-tetraazacyclododecane-1,4,7,10-tetraacetic acid (DOTA-NCS, Macrocyclics, Inc., Dallas, TX) to yield a CNT-DOTA construct in metal-free conditions at pH 9.5 (adjusted with 1 M metal free carbonate solution) for 40 minutes at room temperature at a stoichiometry of 2∶1 (DOTA-NCS to amine). The product was purified using a 10 DG gel permeation column (BioRad, Hercules, CA) with metal-free water (MFW, Purelab Plus System, U.S. Filter Corp., Lowell, MA) as the mobile phase. The 10 DG column was rendered metal free by washing with 30 mL of 10 mM EDTA followed by rinsing with 60 mL of MFW. The product was lyophilized to yield a solid that was the CNT-DOTA construct (Figure 1, compound **3**). Raman spectroscopy was employed to characterize **3** (as described above). AFM images were obtained using a Veeco Dimension 3100 (Woodbury, NY) operated in the tapping mode with a scanning frequency of 84 Hz and a scan rate of 0.500 Hz. Cantilever tips were supplied from Vecco probes (force constant of 3 k/m). Samples were spin coated onto a freshly cleaved mica surface. TEM images were obtained using a Philips EM-201 (Amsterdam, Netherlands) with 1 nm resolution, HV = 80 kV. Samples are adsorbed onto plasma-treated formvar-coated copper grids. Ultraviolet-visible (UV-Vis) spectroscopic and chromatographic characterization of **3** was performed by gel permeation HPLC using a Beckman Coulter System Gold Bioessential 125/168 diode array detection system (Beckman Coulter, Fullerton, CA) equipped with an in-line Jasco FP-2020 fluorescence detector (Tokyo, Japan). Briefly, 0.020 mg of **3** dissolved in *N,N*-dimethylformamide (DMF, Sigma Biotech grade 99.9+%) was chromatographed with a stationary phase of PLgel MIXED-A column (300 mm×7.5 mm) (Polymer Laboratories, Amherst, MA) and a DMF mobile phase at 1 mL/min at ambient temperature. The extent of DOTA substitution per mass of CNT in compound **3** was determined using the spectrophotometric method [13] of Dadachova *et al.* The number of primary amines that remained after appending the DOTA-NCS was again assayed using the Sarin assay [12]. The mass of CNT was determined using UV-Vis spectroscopy (at 600 nm) to measure the CNT concentration from the linear region of a standard curve of absorbance at 600 nm versus different concentrations of CNT.

Yttrium-86 was produced in the Memorial Sloan-Kettering Cancer Center cyclotron core facility *via* the (p,n) nuclear reaction on an enriched strontium-86 target, and chemically separated from the target using ion chromatographic techniques [14], [15]. Activity was assayed in a Squibb CRC-15R Radioisotope Calibrator (E.R. Squibb and Sons, Inc., Princeton, NJ) set at 711 and dividing the displayed activity value by 2.

The ^86^Y-CNT construct (Figure 1, compound **4**) was prepared by adding 296 MBq (8 mCi) of acidic ^86^Y chloride to 0.150 mg of CNT-DOTA (compound **3**) (10 g/L) in MFW and 0.050 mL of 3 M ammonium acetate (Aldrich Chemical Co., Milwaukee, WI) to yield a pH 5.5 solution. The reaction mixture was heated to 60°C for 30 min. and then purified by size exclusion chromatography using a P6 resin (BioRad) as the stationary phase and 1% human serum albumin (HSA, Swiss Red Cross, Bern, Switzerland) in 0.9% NaCl (Abbott Laboratories, North Chicago, IL) as the mobile phase.

The control construct was a mixture of ^86^Y-DOTA and CNT-amine (compound **2**). This mixture was prepared by the adding 37 MBq (1 mCi) of acidic ^86^Y chloride to 0.5 mg (10 g/L in MFW) of 1,4,7,10-tetraazacyclododecane-1,4,7,10-tetraacetic acid (DOTA, Macrocyclics, Inc.) and 0.050 mL of 3M ammonium acetate to yield a pH 5.5 solution. The reaction mixture was heated to 60°C for 30 min. and then purified by size exclusion chromatography as described above. 0.150 mg of **2** in MFW was added to the ^86^Y-DOTA product and mixed.

A small aliquot of each final product was used to determine the radiochemical purity by ITLC-SG using silica gel impregnated paper (Gelman Science Inc., Ann Arbor, MI). The paper strips were developed using two different mobile phases. Mobile phase I was 10 mM EDTA and II was 9% NaCl/10 mM NaOH. The strips were spotted, developed, dried and counted intact using an Ambis 4000 gas ionization detection system (Ambis Inc., San Diego, CA).

### Synthesis and Characterization of ^111^In-CNT

The analogous ^111^In-CNT construct (compound **5**) was prepared by adding 111 MBq (3 mCi) of acidic ^111^In chloride (Perkin Elmer, N. Bellerica, MA) to 0.150 mg of **3** (10 g/L) in MFW and 0.050 mL of 3 M ammonium acetate to yield a pH 5.5 solution. The reaction mixture was heated to 60°C for 30 min. and then purified by size exclusion chromatography using a P6 resin as the stationary phase and 1% HSA in 0.9% NaCl as the mobile phase. A small aliquot of the final product was used to determine the radiochemical purity by ITLC-SG using the methods described above. Combined spectroscopic, radiographic, and chromatographic characterization of the CNT construct was performed by reverse phase HPLC, using a Beckman Coulter System Bioessential 125/168 diode array detection instrument equipped with an in-line γ-RAM Model 3 radioactivity detector (IN/US, Tampa, FL). There was a delay of 0.3 min. that corresponded to the time to transit from the diode array detector to the downstream radionuclide detector. Compound **5** (0.010 mg) was analyzed using a Gemini (Phenomenex, Torrence, CA) 5u reverse phase C18 column (250×4.6 mm) to chromatograph these molecules with a 0 to 100% gradient of 0.1M tetraethylammonium acetate (Aldrich), pH 6.5 and acetonitrile (Aldrich) at a flow rate of 1 mL/min.

### Experimental Design

Ten male athymic nude mice (Taconic, Germantown, NY), 10–12 weeks old, were separated into three groups. Group I (n = 4) received an intravenous (i.v.) injection of 6.7 MBq (0.18 mCi) and 0.012 mg **4** in 0.20 mL *via* the retroorbital sinus. Group II (n = 3) received an intraperitoneal (i.p.) injection of 6.7 MBq and 0.012 mg **4** in 0.20 mL. Group III (n = 3) received an intravenous (i.v.) injection of 13.3 MBq (0.36 mCi) ^86^Y-DOTA+0.015 mg of **2** in 0.20 mL *via* the retroorbital sinus. All animals were imaged by PET on day 1 at 3 hours post-injection; group I and II animals were imaged by CT at this time as well, while still under anesthesia. On day 2, 24 hours post-injection, Group I and II animals were imaged by PET. For all *in vivo* experiments housing and care were in accordance with the Animal Welfare Act and the Guide for the Care and Use of Laboratory Animals. The animal protocols were approved by the Institutional Animal Care and Use Committee (IACUC) at Memorial Sloan-Kettering Cancer Center.

### CT Imaging

CT imaging was performed using the CT component of the X-SPECT (Gamma Medica, Northridge, CA) a dedicated small-animal SPECT-CT scanner for non-invasive, ultra-high-resolution imaging *in vivo* of single-photon-emitting radiotracers and ultra-high-resolution CT scans for anatomic registration. The imaging times for the CT studies were 10 min. with a resolution of ≈0.100 mm. These CT imaging studies require animals to be fully anesthetized using isofluorane anesthesia.

### Micro-PET Imaging

The microPET Focus™ 120 (CTI Molecular Imaging, Inc., Knoxville, TN) is a dedicated small-animal scanner for imaging PET radiotracers. An energy window of 350–750 keV, a coincidence timing window of 6 nsec, and an acquisition time of 10–20 min. were used. The resulting list-mode data were sorted into 2D histograms by Fourier re-binning and transverse images reconstructed by filtered back-projection into a 128×128×95 matrix. The reconstructed spatial resolution is 2.6 mm full-width half maximum (FWHM) at the center of the field of view. The image data were corrected for non-uniformity of response of the microPET™ (i.e. were normalized), deadtime count losses, physical decay to the time of injection, and the ^86^Y positron branching ratio but no attenuation, scatter, or partial-volume averaging correction was applied. An empirically determined system calibration factor (i.e. µCi/mL/cps/voxel) for mice was derived by imaging a mouse-size cylinder containing ^18^F uniformly dispersed in water and used to convert voxel count rates to activity concentrations. The resulting image data were then normalized to the administered activity to determine by region-of-interest analysis the percent of the injected dose per gram (%ID/g) of tissue corrected for radioactive decay to the time of injection.

### Data Analysis

AsiPRO VM 5.0 software (Concorde Microsystems, Knoxville, TN) was used to perform image and region of interest (ROI) analyses with the PET and CT datasets. For the ROI analyses, a minimum of 3 regions per tissue per animal were collected and the average %ID/g and standard deviation determined. The average %ID/g tissue per animal was used to determine an average %ID/g per tissue per group and the standard deviation within the group values was calculated. Standards of each injected formulation were counted to quantify the %ID/g. Unpaired two-tailed t-tests were performed to assess temporal differences of tissue activity. Prism software (Graphpad Software, Inc., San Diego, CA) was used for statistical analyses and plotting data. TEM image analysis was performed using ImageJ software (NIH, http://rsb.info.nih.gov/ij/). AFM image analysis was carried-out using Nanoscope II software from Digital Instruments.

### Biodistribution Data

Animals were sacrificed at 24 h post-injection and the kidneys, liver and spleens were harvested according to approved IACUC institutional protocols. Tissue samples were weighed and counted in a Packard Cobra γ-counter (Packard Instrument Co., Inc., Meriden, CT) using a 315–435 KeV window. Standards of each injected formulation were counted to determine the %ID/g.

### Blood Clearance and Excretion of ^111^In-CNT

In another biodistribution study, 0.007 mg of **5** (specific activity 37 GBq/g (1Ci/g)) were injected i.v., *via* the retroorbital sinus, per BALB/c mouse (female, 8–12 weeks old, Taconic Farms, NY) and the mice were divided into 5 groups of n = 3 per group. One group of mice was sacrificed at each of the following time points 1, 24, 96, 216 and 360 hours and tissue samples including blood, brain, lung, heart, adipose tissue, liver, kidney, spleen and femur were harvested, weighed and counted using a Packard Cobra Gamma counter using the 15–550 KeV window. The %ID/g was determined by measuring the activity of an aliquot of each construct injectate. Urine samples were collected from mice 1 hour post injection. Activity was detected and the urine samples were analyzed using the ITLC method described above.

## Results

### The synthesis, radiolabeling, and characterization of the CNT constructs

**Figure 2:**
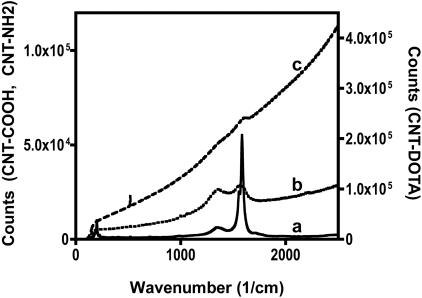
Raman spectra measured with a 488 nm laser. (a) acid-oxidized and purified CNT, compound 1; (b) sidewall amine-functionalized CNT, compound 2; (c) DOTA-functionalized CNT, compound 3. The spectra for 1 and 2 are plotted on the left axis (0-120,000), and 3 on the right axis (0–450,000).

**Figure 3:**
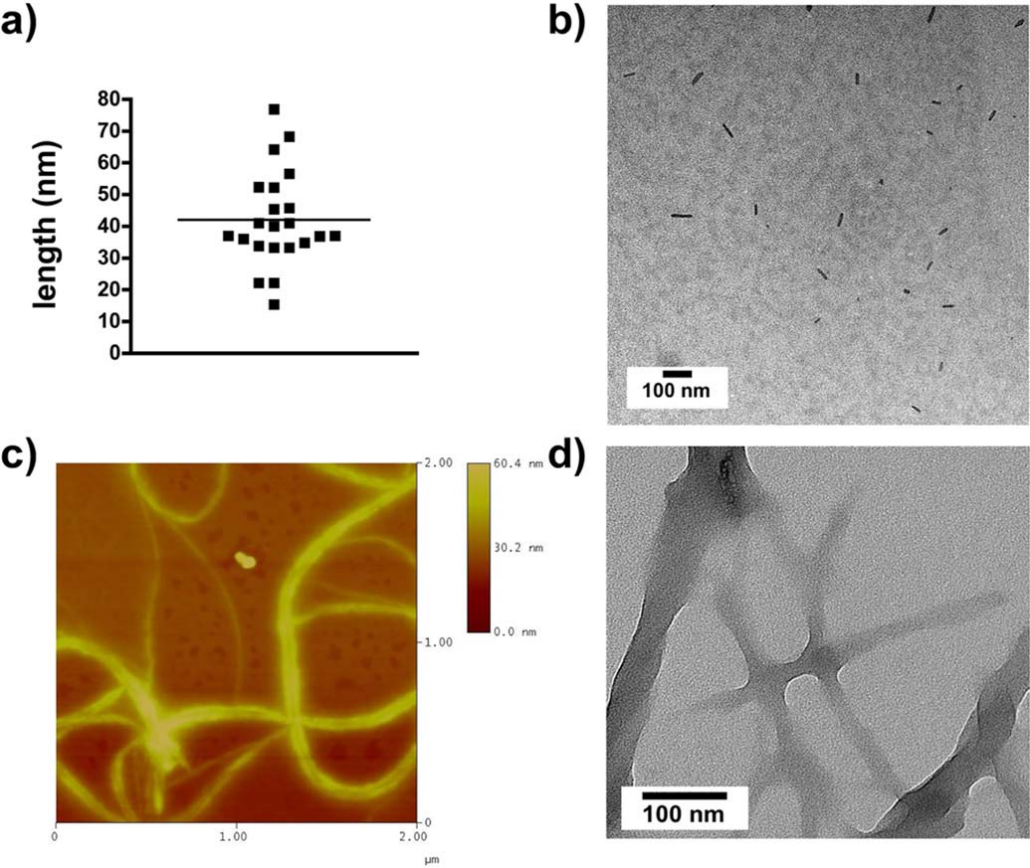
Microscopic images of 3 provide data to evaluate the (a) size distribution of 22 individual DOTA-CNT obtained from (b) a representative TEM image of solubilized 3 (scale bar = 100 nm); (c) AFM image of the height (scale bar = 0–60 nm) and (d) TEM image (scale bar = 100 nm) of representative DOTA-CNT bundles.

**Figure 4:**
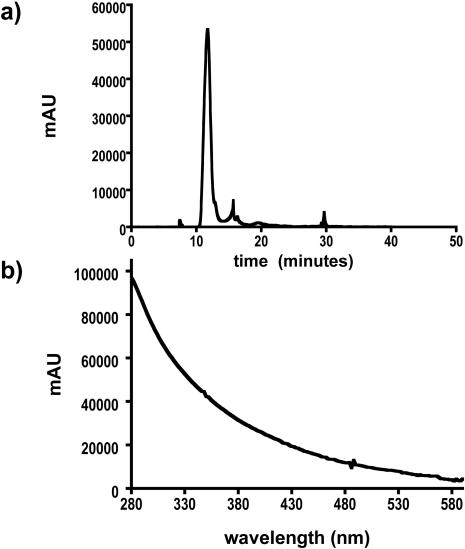
Gel permeation HPLC chromatograph of compound 3 (0.020 mg) dissolved in DMF on a stationary phase of PLgel MIXED-A column with DMF mobile phase at 1 mL/min at ambient temperature. (a) Chromatograph is shown with absorbance at 330 nm. (b) The UV-VIS absorbance spectrum of the peak at 11.7 min.

Pristine CNT were purified and shortened by digestion in dilute nitric acid and sonication. The product of this process, **1**, was carboxy-functionalized CNT. The Raman spectrum of this product is shown in Figure 2a. The radial breathing mode (RBM) feature at 200 cm^−1^ yields a CNT diameter of 1.1 nm and further confirmation of the product identity is provided by the 1340 cm^−1^ disorder band (D band) and the sharp 1556 and 1584 cm^−1 ^tangential mode (G band) resonances [16], [17]. These CNT (compound **1**) were functionalized and solubilized by the covalent sidewall attachment of pendant primary amines to yield compound **2**. The Raman spectrum of **2** (Figure 2b) was characterized by the loss of the RBM feature and a rising baseline due to increasing luminescence resulting from the enhanced dispersion of this product in solution [18]. The amine content of the construct was quantified using the Sarin assay [12], which yielded a value of 1.76 mmol amine/g CNT. Subsequently, these amines were reacted with DOTA-NCS to yield the CNT-DOTA construct (compound **3**). Raman characterization of **3** (Figure 2c) confirmed the enhanced solubility (∼10 g/L) of this construct by the rising baseline [18], which partially masks the D and G bands. Transmission electron and atomic force microscopic images (Figure 3) of **3** are presented to demonstrate the composition, identity and purity of the DOTA-functionalized CNT product. Figure 3a displays the results of an image analysis of the TEM data of a sampling of 22 ‘unbundled’ **3** in Figure 3b. This data shows that the mean±standard deviation CNT length distribution of **3** was 42±17 nm (*n* = 22). The AFM and TEM images of **3** in Figures 3c and d, respectively, show bundles of **3** with varying thickness (approximately 1–20 nm) and lengths and the absence of carbonaceous or nanoparticulate contaminants. A gel permeation HPLC chromatograph of **3** is shown in Figure 4 with a major absorbance peak at 11.7 min and the corresponding UV-Vis absorbance spectrum of that peak demonstrating the characteristic CNT spectral feature. This gel permeation chromatograph further demonstrates the purity of **3**, notably by the absence of the later eluting amorphous carbon and nanoparticulate species [19], which are highly fluorescent species [18] and that were not detected using the in-line fluorescence detector. The DOTA content of **3** was determined using a lead arsenazo assay [13], which yielded a value of 0.30 mmol DOTA/g CNT; in addition **3** still contained some unreacted amines assayed to be 0.27 mmol amine/g CNT. Construct **3** was labeled with “no carrier added” ^86^Y chloride [14], [15] and purified by size exclusion chromatography. The radiochemical purity of compound **4** was determined to be 90% by ITLC methods and had a specific activity of 555 GBq/g (15 Ci/g). The control construct was a mixture of ^86^Y-DOTA+**2**. The ^86^Y-DOTA was 95% radiochemicaly pure as determined by ITLC-SG.

**Figure 5:**
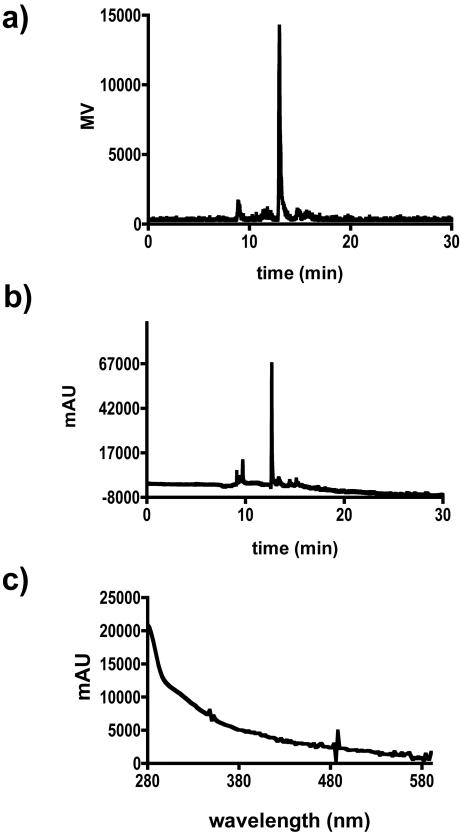
Reverse phase HPLC chromatograph of ^111^In-CNT, compound 5 (0.010 mg); (a) Radionuclide trace of 5; (b) Corresponding chromatograph with absorbance at 350 nm (*n.b.*, there was a 0.3 min delay between the detectors); (c) The UV-VIS absorbance spectrum of the peak at 12.7 minutes in panel b.

An^ 111^In-CNT construct (compound **5**) was similarly prepared and purified. This radiochemical analog was used in ancillary animal experiments to measure blood clearance and excretion *in vivo* over an extended period of time due to it's longer half-life. ^111^In has been extensively utilized as a surrogate radionuclide for yttrium chemistry in pre-clinical and clinical studies. Compound **5** also served to validate the ITLC-SG methods for determining radiochemical purity. The ^111^In-CNT product was determined to be 90% by the ITLC methods described above, similar to the observed ITLC-SG purity of **4**. A reverse phase HPLC analysis of **5** confirmed the co-elution of CNT and radioactivity (Figure 5). The diode array detected a sharp absorbance peak at 12.7 minutes (Figure 5b) that was attributed to the ^111^In-CNT product and confirmed by the characteristic CNT spectral signature (Figure 5c). The corresponding radioactivity trace (Figure 5a) revealed a sharp peak at 13.0 minutes that contained 90% of the eluted radioactivity activity and after correction for the delay between detectors, was assigned to the ^111^In-CNT product. Two uniquely different chromatographic methods were developed and yielded the same result for the radiochemical purity - the reverse phase HPLC method and the isocratic ITLC method. The reverse phase HPLC data also correlated the radioactivity with the CNT, thus validating the use of ITLC to rapidly and quantitatively determine radiochemical purity and identity of these constructs.

### The biodistribution of the radiolabeled construct and the control construct

The ten mice were separated into three groups, each mouse in groups I and II received 0.012 mg of **4** labeled with 6.7 MBq of ^86^Y, administered i.v. or i.p., respectively. Animals in group III (control group) each received a physical mixture of 13.3 MBq of ^86^Y-DOTA and 0.015 mg of **2** *via* i.v. injection. We evaluated the biodistribution of **4** *in vivo* by serial PET and then performed anatomic imaging using a dedicated small-animal CT scanner. Software fusion of the resulting PET and CT images was carried out to combine the images. The imaging results were confirmed by sacrificing the animals and harvesting, weighing, and counting the activity in the organs.

**Figure 6:**
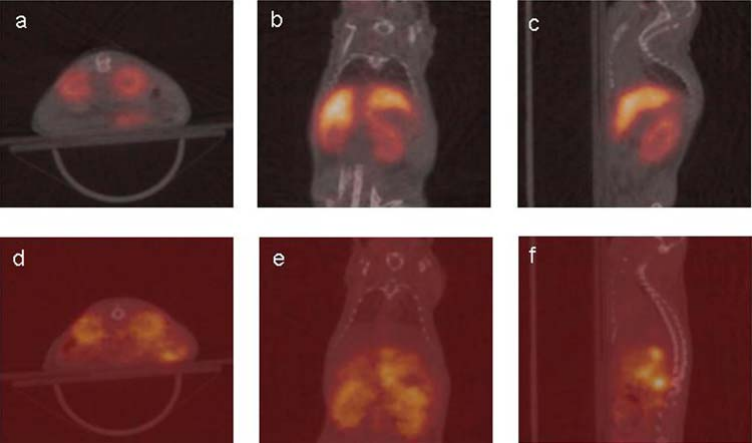
Fused PET and CT images at 3 hours post-injection of ^86^Y-CNT, compound 4, showing a transverse (z-direction, images a and d), a coronal (y-direction, images b and e), and a sagittal (x-direction, images c and f) slice of one representative animal from group I (i.v., a, b and c) and one from group II (i.p., d, e and f).

PET images of animals in groups I and II at 3 hours post-injection clearly demonstrated accumulation of activity in the kidneys, spleen and liver (Figure 6). The kidney images showed uptake primarily in the renal cortex, while the renal medulla was devoid of activity. There was no blood-pool activity observed in these images. In group I, the ^86^Y-CNT construct appeared to avidly localize in the spleen and liver. In group II, spleen and liver uptake was significantly lower and a diffuse activity was evident from the i.p. cavity suggesting a relatively slow egress from the i.p. compartment into the vascular compartment. After 24 hours there was no significant difference in the accumulated activity in the spleen and liver within each group compared to the 3 hour time-point. Additionally, the kidneys of the animals in groups I and II had begun to clear the constructs. These data indicate that the clearance from the spleen and liver is slower than from the kidneys. The radioactivity in the liver of the group I animals was still significantly higher than the group II animals at both time-points evaluated. As expected for a stably chelated metal-ion species with a relatively small molecular weight, the control mixture cleared completely from group III within 3 hours. The imaged radioactivity was located primarily in the bladder (∼1 %ID/g at 3 hours) and was consistent with urinary excretion of small molecules (data not included).

**Figure 7:**
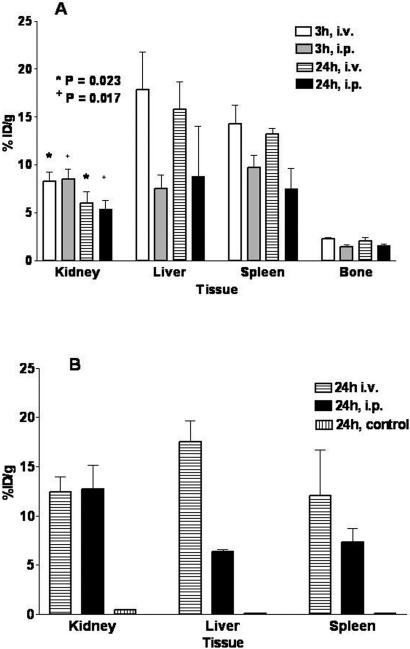
Biodistribution data obtained from the PET imaging experiment and from tissue harvest. (a) Plot of the 2-dimensional ROI data for all group I and II animals at 3 and 24 hours. Three regions per tissue per animal were collected and the average %ID/g and standard deviation determined. The average %ID/g tissue per animal was then used to determine an average %ID/g per tissue per group and the standard deviation within the group values was also calculated. (b) Plot of the 24 hour biodistribution data obtained from tissue harvest, weighing and counting of organs from group I, II, and III mice.

We performed region of interest (ROI) analysis of the PET images in each of the group I and II animals to extract a decay-corrected activity concentration in various organs. Data (Figure 7a) are reported as the mean±standard deviation %ID/g. The kidneys had 8.30±0.92 and 8.50±1.05; the liver had 17.8±3.95 and 7.54±1.38; the spleen had 14.3±2.0 and 9.7±1.3; and the bone (femur and spine) had 2.26±0.14 and 1.44±0.23 %ID/g at 3 hours post-injection in the group I and II animals, respectively.

At 24 hours post-injection, the kidneys had 5.96±1.23 and 5.42±0.85; the liver had 15.8±2.90 and 8.83±5.17; the spleen had 13.2±0.60 and 7.51±2.12; and the bone had 2.02±0.36 and 1.56±0.17 %ID/g in the group I and II animals, respectively. The accumulated activity in the kidneys cleared significantly in group I (P = 0.023) and group II (P = 0.017) over this 21-hour period. However, statistical analysis showed that the activity accumulated in the spleen, liver and bone was not clearing over this period of time.

Residual ^86^Y-CNT activity that was slowly exiting the i.p. compartment yielded poorer image contrast in the group II mice. Interestingly, the construct activity that did exit the i.p. cavity had the same kidney uptake as the i.v. administered construct at both timepoints, but very different liver and spleen accumulation. The %ID/g activity in the livers of group II animals is 42% of that in the group I animals at 3 hours post-injection, and 56% of that in the group I animals at 24 hours post-injection. It appeared that the equilibration process is slow and the liver activity increases with time in the group II animals.

Biodistribution data (Figure 7b) obtained from tissue harvest at 24 hours was in good agreement with the ROI data. The spleen showed the same differential accumulation as the liver as a function of i.p. *versus* i.v. administration. Group III control animals showed no organ-specific uptake of ^86^Y with only small amounts of activity (0.1 to 1 %ID/g) in the kidney, liver and spleen. The post-mortem biodistribution data and the 3-hour PET images showed this control mixture rapidly cleared from the group III mice.

The whole-body PET images indicated that the major sites of accumulation of activity resulting from the administration of ^86^Y-CNT were the kidney, liver, spleen, and to a much less extent the bone. The % ID/organ values for kidney, liver, and spleen, 24 hours post- i.v. injection, were 5.96±1.20, 15.2±1.54, and 0.82±0.04, respectively. The corresponding % ID/organ values for kidney, liver, and spleen, 24 hours post- i.p. injection, were 5.48±0.61, 4.46±1.08, and 0.60±0.09, respectively. Whole-body activity was decreasing over this 24 hour period of time, as we could account for only about 20% of the total i.v. administered activity in the group I animals and about 11% of the total activity in the group II animals. In another biodistribution experiment, ^111^In-CNT was injected into mice and ITLC analysis of urine samples obtained 1 hour after injection was consistent with ^111^In-CNT being excreted.

**Figure 8:**
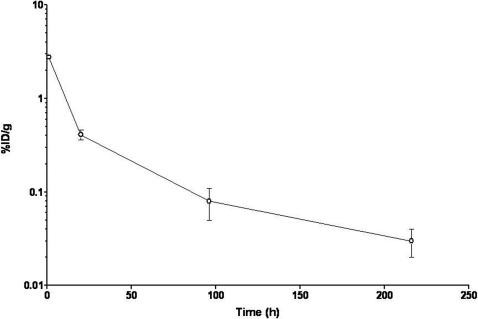
Plot of the blood clearance of ^111^In-CNT, compound 5, following i.v. injection (*n* = 3 per group).

The clearance of radiometal-labeled CNT constructs from the blood compartment is rapid. This was observed in this study (no blood pool activity at 3 h) and quantitatively measured in another biodistribution study using the longer half-lived ^111^In. Briefly, compound **5** was injected into mice using the retroorbital sinus route and venous blood samples were obtained 1, 20, 96, 216, and 360 hours after injection from groups of three animals per timepoint. The data are shown in Figure 8. After 1 hour 2.76±0.14 %ID/g was still in the blood and after 20 h only 0.41±0.05 %ID/g was in circulation. The experiment was continued for 15 d at which time no activity was found in circulation. Additional ^111^In-CNT biodistribution data are presented in a study of lymphoma tumor targeting with an antibody-functionalized, radiolabeled carbon nanotube [20]. Tissue harvest indicated that the major sites of ^111^In accumulation were the kidney, liver, spleen, and to a much less extent the bone, similarly to the ^86^Y accumulation. ^111^In cleared the kidneys more rapidly than the spleen and liver.

## Discussion

The interface of CNT with biological systems is a key issue that can be addressed in part by determining the pharmacokinetic profile of these unique materials. Where do functionalized-CNT distribute *in vivo* following administration and how do they clear are the questions that were posed in this study. Published reports of the biodistribution of soluble, sidewall-functionalized, radiolabeled CNT in murine models [6], [7], [20] were very different. The first two biodistributions were based on tissue harvest and did not contain an imaging component. Wang et al. [6] used ^125^I-CNT (*l*∼300 nm) where the CNT were hydroxyl-functionalized. They reported the ^125^I cleared the blood and accumulated in the stomach, kidneys and bone; stomach accumulation is not unusual for free ^125^I. Bone and kidney had accumulated activity that persisted for up to 6 days. 80% of the activity cleared the animals over an 11 day period. They did not report notably different tissue accumulations when the construct was administered i.v. or i.p. Singh et al. [7] used CNT (*l*∼300–1000 nm) that were amine-functionalized, modified with DTPA anhydride, and then labeled with ^111^In. The ^111^In cleared the blood compartment rapidly (within 3 hours) and surprisingly there was no significant tissue uptake. McDevitt et al. [20] reported the selective targeting of tumor *in vivo* using tumor-specific antibodies that were covalently-appended to a soluble, ^111^In-radiolabeled CNT construct relative to controls of a i) non-targeting antibody-appended CNT construct; ii) a non-targeting CNT construct, and iii) antibody alone. Clearance of the non-targeting, radiolabeled CNT construct from the blood compartment was rapid. Tissue harvest indicated that the major sites of ^111^In accumulation were the kidney, liver, spleen, and to a much less extent the bone. ^111^In cleared the kidneys more rapidly than the spleen and liver. Radioactive CNT was in the urine samples collected from mice 1 hour post injection. The covalent attachment of antibodies to the CNT scaffold dramatically altered the kidney biodistribution and pharmacokinetics relative to the non-targeting, radiolabeled CNT construct.

Small globular molecules (<25 kDa) are typically filtered by the kidney but may also be reabsorbed subsequently in the tubule. Large globular molecules usually bypass clearance through the kidney, CNT may not behave similarly due to their large aspect ratio, unlike globular proteins. Singh *et al.* [7] observed rapid and complete renal clearance of their injected material, which is difficult to explain given the 25 kDa cut-off of the glomerulus and the 0.1 to 1 mDa (approximate) weight range of the CNT materials.

Compound **3**, the DOTA-CNT starting material, used to prepare the radiolabeled constructs **4** and **5**, was microscopically, spectroscopically, and chromatographically characterized to confirm the identity and purity of this reagent. These were “short” tubes with mean lengths of 42±17 nm (n = 22) resulting from the acid digestion and sonication processing steps [21]–[23]. The AFM and TEM images of **3** show bundles of varying thickness and lengths with no carbonaceous or particulate contaminants. Gel permeation chromatography of **3** identified a major absorbance peak that had the spectral signature of CNT and which did not exhibit any carbonaceous or particulate contaminants. Compounds **4** and **5** were characterized by traditional radiochemical ITLC methods which were validated using radio-HPLC methods. The radioactivity (^86^Y or ^111^In) was clearly associated with the CNT, which is not surprising given the stability of the DOTA-metal-ion complex and the thiourea linkage [24] between the DOTA and the CNT. Chelates such as DTPA anhydride were significantly less stable than DOTA or backbone-modified DTPA moieties [25]–[27].

Compounds **4** and **5**, that we report herein, were fully water-soluble and were stable in the 1% human serum albumin (HSA) injection vehicle and stable in biological fluids [20]. We were not surprised that high molecular-weight, water-soluble compounds that are CNT-based were accumulated in the liver and spleen and also in the kidney. Previous work [6], [20] also observed accumulation of their CNT constructs in the kidneys. Hydrophilic fullerenes were reported [28], [29] to partition into liver and spleen, but exhibit little-to-no accumulation in kidney, presumably because of their smaller size and shape. It was surprising that the constructs reported by Singh *et al.* clear the mouse entirely within a few hours and do not accumulate anywhere.

A pharmacokinetic report [8] of unmodified CNT (*l*∼300 nm) suspended in a surfactant and injected i.v. into rabbits employed the inherent near-infrared fluorescence of CNT for detection in blood and tissue samples. They report a rapid blood clearance (half-life of 1.0 h) and were only able to detect CNT in the liver, but not in any other tissue sample examined (0.020 mg/kg was administered). The materials that they examined were unmodified, insoluble CNT and these may exhibit very different properties than the modified, soluble CNT constructs used in this and the other three studies [6], [7], [20]. Others have coated CNT with non-covalently attached radiolabeled- and RGD-labeled surfactants and administered (0.05 mg/kg) to mice [30]. This type of construct is more similar to the unmodified CNT [8] in that the CNT was dispersed in a detergent solute to facilitate solubilization. These molecules were fundamentally very different than the robust, covalently assembled constructs reported herein and elsewhere [6], [7], [20], since the radiolabel and the RGD were not attached to the CNT. Unfortunately, they did not report [30] the biodistribution of the radiolabeled surfactant alone, so that one can compare its biodistribution to that of the construct nor of a control construct (e.g., labeled with a non-specific control peptide sequence). Thus the biodistribution can not be inferred with certainty.

In this biodistribution study we performed an *in vivo* imaging study of soluble, sidewall-functionalized, radiolabeled CNT constructs in mice utilizing PET. By employing PET imaging (and tissue harvest), we were able to measure accumulation and clearance in groups of living animals over time by observing each subject. The radioactivity from these compounds also accumulated in bone and kidney, was cleared rapidly from the blood, and was excreted in the urine. The spleen and liver accumulation, and the differential clearance from the i.p. cavity, may result from a different hydrophobicity of the hydroxylated [6] *versus* chelate/amine functionalized CNT. While our CNT constructs were processed similarly to those in reference 7, in that paper the isotope completely cleared the blood and body rapidly.

We speculated that the CNT most likely cleared *via* i) longitudinal filtration [31] through the renal glomeruli, which has a pore size of approximately 30 nm diameter or ii) active secretion into the tubular lumen. Accumulation of CNT in the kidneys, in particular in the renal cortex region where filtration occurs, was clearly shown in Figure 6a-c. The effect of molecular shape, charge and size on kidney clearance has been investigated using different macromolecular solutes [32], [33]. The frictional ratio of the molecule plays an important role as does the shape in crossing the glomerular barrier. The negatively charged capillary endothelium, anionic glomerular basement membrane, and the anionic podocyte cell coating might provide a charge gradient that facilitates transit of amine-functionalized CNT from the blood to the urine [31]. Differences in CNT construct purity, chemistry, charge, and flexibility could alter the biodistribution. The covalent attachment of antibody molecules to the CNT dramatically altered the kidney biodistribution and pharmacokinetics compared to ^111^In-CNT in tumor-bearing *versus* non-tumor-bearing mice [20]. PET/CT provides a comprehensive measure of *in vivo* behavior to which the physicochemical properties of each construct can be compared.

A comparison of the PET images obtained at 3 and 24 hours demonstrated that the diffuse activity imaged in the i.p. cavity of the group II animal persists over the 24 hour period of the experiment and the liver activity was less than that in the group I animals. This observation was interesting since large globular proteins, for example IgG (∼150 kDa), injected into the i.p. cavity of a mouse redistributed rapidly and reached equilibrium in the blood within 5 to 6 hours (data not shown). The mean ^86^Y-CNT construct size was approximately 100 kDa, but obviously it did not behave as a globular protein. The persistence of radioactivity in the i.p. cavity seemed to minimize liver and spleen accumulation.

The mass amounts of CNT construct that were used per animal in this study (0.6 mg/kg) were in the same range as those that were employed in a study with radio-labeled, targeting-antibody CNT constructs in mice [20]. The amount of activity accumulated in the organs of these animals after 24 hours (The % ID/organ values for kidney, liver, and spleen, post- i.v. injection, were 5.96±1.20, 15.2±1.54, and 0.82±0.04, respectively; the corresponding values post- i.p. injection, were 5.48±0.61, 4.46±1.08, and 0.60±0.09, respectively.) does not pose a problem in translating these constructs to other studies. Further, we have demonstrated that appropriately designed targeting diagnostic agents based on a CNT platform might further alter liver, kidney and spleen accumulation/clearance [20]. The rapid blood clearance of these nanomaterials could prove beneficial when using short half-lived radionuclides in diagnostic applications, as prolonged persistence of blood-pool activity following antibody-construct administration obscures target imaging due to a low signal-to-background ratio. The mechanism of excretion from the kidneys must also be further examined to better understand the construct parameters that affect clearance.

### Conclusion

The whole-body PET images indicated that the major sites of accumulation of radioactivity resulting from the administration of soluble, sidewall-functionalized ^86^Y-CNT were the kidney, liver, spleen, and to a much less extent the bone. PET data provides extremely sensitive, quantitative, and functional information that is different from that obtainable with other largely anatomical imaging modalities and CT was performed to confirm anatomical assignments. The presence of activity in the renal cortex where glomerular filtration occurs was clearly shown in Figure 6 and correlates with the radioactivity found in the urine. Furthermore, the rapid blood clearance that was observed can be beneficial in the use of short-lived radionuclides in diagnostic applications by reducing the background activity compared to target tissue activity. The mass amounts of soluble CNT construct that were used per animal (0.6 mg/kg) were 30-fold higher than those applied in a study with un-functionalized CNT [8] and point-out the utility of functionalized-CNT which not only improves solubility but acts as a scaffold that can bear many copies of an appended moiety. The CNT compounds in this study were very short-length and chemically and radiochemically pure. The extensive physical and chemical characterization studies yielded a clear description of the identity and purity of these CNT compounds and proved useful in better understanding their pharmacokinetic profile. Furthermore, the careful radiochemical characterization and experimental controls better correlated the observed pharmacokinetic results with the composition and identity of the material injected.

We believe that the results described herein encourage further study of CNT constructs in vivo. This soluble, sidewall-functionalized CNT platform rapidly cleared the blood compartment and exhibited some distribution to the expected clearance sites. We have also demonstrated the utilization of an antibody-modified-CNT construct in targeting human tumor in a murine model [20]. The emerging field of nano-medicine will find applications for many such CNT-based molecular constructs based upon this synthetic and imaging approach.

## References

[1] Dresselhaus MS, Avouris P (2001). Introduction to carbon materials research.. Topics in Applied Physics.

[2] in het Panhuis M (2003). Vaccine delivery by carbon nanotubes.. Chem Biol.

[3] Cherukuri P, Bachilo SM, Litovsky SH, Weisman RB (2004). Near-infrared fluorescence microscopy of single-walled carbon nanotubes in phagocytic cells.. J Am Chem Soc.

[4] Shi Kam NW, Jessop TC, Wender PA, Dai HJ (2004). Nanotube Molecular Transporters: Internalization of Carbon Nanotube-Protein Conjugates into Mammalian Cells.. J Am Chem Soc.

[5] Pantarotto D, Briand JP, Prato M, Bianco A (2004). Translocation of bioactive peptides across cell membranes by carbon nanotubes.. Chem Commun.

[6] Wang H, Wang J, Deng X, Sun H, Shi Z (2004). Biodistribution of carbon single-wall nanotubes in mice.. J Nanosci Nanotechnol.

[7] Singh R, Pantarotto D, Lacerda L, Pastorin G, Klumpp C (2006). Tissue biodistribution and blood clearance rates of intravenously administered carbon nanotube radiotracers.. Proc Natl Acad Sci U S A.

[8] Cherukuri P, Gannon CJ, Leeuw TK, Schmidt HK, Smalley RE (2006). Mammalian pharmacokinetics of carbon nanotubes using intrinsic near-infrared fluorescence.. Proc Natl Acad Sci U S A.

[9] Georgakilas V, Tagmatarchis N, Pantarotto D, Bianco A, Briand JP (2002). Amino acid functionalisation of water soluble carbon nanotubes.. Chem Commun.

[10] Pantarotto D, Partidos DC, Graff R, Hoebeke J, Briand JP (2003). Synthesis, structural characterization, and immunological properties of carbon nanotubes functionalized with peptides.. J Am Chem Soc.

[11] von Schulthess GK, Steinert HC, Hany TF (2006). Integrated PET/CT: Current Applications and Future Directions.. Radiology.

[12] Sarin VK, Kent SBH, Tam JP, Merrifield RB (1981). Quantitative monitoring of solid-phase peptide synthesis by Ninhydrin reaction.. Anal BioChem.

[13] Dadachova E, Chappell LL, Brechbiel MW (1999). Spectrophotometric method for determination of bifunctional macrocyclic ligands in macrocyclic ligand-protein conjugates.. Nucl Med Bio.

[14] Finn RD, McDevitt M, Ma D, Jurcic J, Scheinberg D (1999). Low Energy Cyclotron Production and Separation of Yttrium-86 for Evaluation of Monoclonal Antibody Pharmacokinetics and Dosimetry. In: Duggan JL, Morgan IL, editors. Application of Accelerators in Research and Industry. Woodbury, NY: American Institute of Physics. pp..

[15] Lovqvist A, Humm JL, Sheikh A, Finn RD, Koziorowski J (2001). PET imaging of (86)Y-labeled anti-Lewis Y monoclonal antibodies in a nude mouse model: comparison between (86)Y and (111)In radiolabels.. J Nucl Med.

[16] Yu Z, Brus L (2001). Rayleigh and Raman scattering from individual carbon nanotube bundles.. J. Phys. Chem. B.

[17] Itkis ME, Perea DE, Jung R, Niyogi S, Haddon RC (2005). Comparison of analytical techniques for purity evaluation of single-walled carbon nanotubes.. J. Am Chem. Soc..

[18] Lin Y, Zhou B, Martin RB, Henbest KB, Harruff BA, Riggs JE, Guo Z-X, Allard LF, Sun Y-P (2005). Visible luminescence of carbon nanotubes and dependence on functionalization.. J. Phys. Chem. B.

[19] Zhao B, Hu H, Niyogi S, Itkis ME, Hamon MA, Bhowmik P, Meier MS, Haddon RC (2001). Chromatographic purification and properties of soluble single-walled carbon nanotubes.. J. Am. Chem. Soc..

[20] McDevitt MR, Chattopadhyay D, Kappel BJ, Jaggi JS, Schiffman SR (2007). Tumor targeting with antibody-functionalized, radiolabeled carbon nanotubes.. J. Nucl. Med..

[21] Huang W, Lin Y, Taylor S, Gaillard J, Rao AM, Sun Y-P (2002). Sonication-assisted functionalization and solubilization of carbon nanotubes.. Nano Letters.

[22] Hu H, Zhao B, Itkis ME, Haddon RC (2003). Nitric acid purification of single-walled carbon nanotubes.. J. Phys. Chem. B.

[23] Arnold K, Hennrich F, Krupke R, Lebedkin S, Kappes MM (2006). Length separation studies of single walled carbon nanotube dispersions.. Phys Stat. Sol. (B).

[24] McDevitt MR, Ma D, Lai LT, Simon J, Borchardt P (2001). Tumor therapy with targeted atomic nano-generators.. Science.

[25] Esteban JM, Schlom J, Gansow OA, Atcher RW, Brechbiel MW (1987). New method for the chelation of indium-111 to monoclonal antibodies: biodistribution and imaging of athymic mice bearing human colon carcinoma xenografts.. J Nucl Med.

[26] Ruegg CL, Anderson-Berg WT, Brechbiel MW, Mirzadeh S, Gansow OA (1990). Improved in vivo stability and tumor targeting of bismuth-labeled antibody.. Cancer Res.

[27] Brouwers AH, van Eerd JE, Frielink C, Oosterwijk E, Oyen WJ (2004). Optimization of radioimmunotherapy of renal cell carcinoma: labeling of monoclonal antibody cG250 with ^131^I, ^90^Y, ^177^Lu, or ^186^Re.. J Nucl Med.

[28] Bullard-Dillard R, Creek KE, Scrivens WA, Tour JM (1996). Tissue sites of uptake of C-14-labeled C-60.. Bioorg Chem.

[29] Cagle DW, Kennel SJ, Mirzadeh S, Alford JM, Wilson LJ (1999). In vivo studies of fullerene-based materials using endohedral metallofullerene radiotracers.. Proc Natl Acad Sci U S A.

[30] Lui Z, Cai WB, He L, Nakayama N, Chen K (2007). In vivo biodistribution and highly efficient tumour targeting of carbon nanotubes in mice.. Nature Nanotech.

[31] Lee CC, MacKay JA, Frechet JM, Szoka FC (2005). Designing dendrimers for biological applications.. Nat Biotechnol.

[32] Ohlson M, Sorensson J, Lindstrom K, Blom AM, Fries E (2001). Effects of filtration rate on the glomerular barrier and clearance of four differently shaped molecules.. Am J Physiol Renal Physiol.

[33] Moffat DB (1981). New ideas on the anatomy of the kidney.. J Clin Pathol.

